# Regulating Together: perspectives on improving emotion regulation in autistic youth

**DOI:** 10.3389/fpsyt.2026.1784591

**Published:** 2026-04-13

**Authors:** Shivali Sarawgi, Sungeun Kang, Lauren M. Schmitt, Debra L. Reisinger, Jennifer R. Ruberg, Rebecca C. Shaffer

**Affiliations:** 1 Division of Developmental and Behavioral Pediatrics, Cincinnati Children’s Hospital Medical Center, Cincinnati, OH, United States; 2 Department of Pediatrics, University of Cincinnati College of Medicine, Cincinnati, OH, United States; 3 Department of Educational Psychology, University of Nebraska-Lincoln, Lincoln, NE, United States; 4 Phelan-McDermid Syndrome Foundation, Osprey, FL, United States

**Keywords:** autism, autism spectrum disorder, emotion dysregulation, emotion regulation, Regulating Together

## Abstract

Addressing etiological and maintaining factors at play in psychopathology is essential for appropriate treatment of symptoms and development of healthy skills. Emotion regulation (ER) is one such factor associated with developmental, familial, social, and academic wellbeing. Autistic youth demonstrate increased difficulties with emotion regulation, which may contribute to several commonly cooccurring psychiatric concerns observed in autism. Despite an increased risk, few interventions target emotion regulation specifically in autistic populations. Importantly, autistic youth may present unique strengths and challenges related to emotion processing, regulatory strategies, and skill building. The goal of this paper is to share clinical perspectives derived from implementation of Regulating Together (RT), a manualized group intervention targeting emotion regulation difficulties in autistic youth, and to use examples from RT to illustrate how ER interventions can address common challenges and draw upon strengths observed in this population. Here, we outline several of these strengths and challenges related to ER difficulties found in the literature and clinical perspectives informed by caregiver reported observations. RT is presented as a model to inform ER intervention approaches to address these challenges and utilize strengths.

## 1 Introduction

Emotion regulation (ER) refers to attempts to influence one’s emotional experience, including type, strength, duration, experience, and expression. Autistic individuals frequently experience challenges in ER, which contribute to elevated emotion dysregulation, a hypothesized transdiagnostic mechanism related to clinically significant behavioral and emotional difficulties (1, 2). Emotion dysregulation has been linked to other concerns, such as depression, anxiety, and aggression (3, 4), which commonly co-occur in autism, as well as increased suicidal ideation, acute care utilization, and difficulty accessing crisis intervention. Interventions designed to support ER skill development, such as building emotional control, have been shown to reduce emotion dysregulation symptoms in individuals with various psychiatric presentations.

In autistic populations, diagnosis-specific characteristics may contribute to difficulties with ER skills and skill development, including differences in response inhibition, emotional insight, and awareness of others’ emotions, as well as social interaction impairments, differences in sensory sensitivity, and difficulty problem solving (5–8). Preliminary empirical evidence suggests explicit teaching and practice with multiple ER strategies can improve ER skills and decrease emotion dysregulation in autistic youth (9). Nevertheless, clinicians may need to adapt commonly utilized ER building strategies and increase supports in treatment to maximize outcomes based on unique strengths and challenges observed in autistic youth.

**Figure 1:**
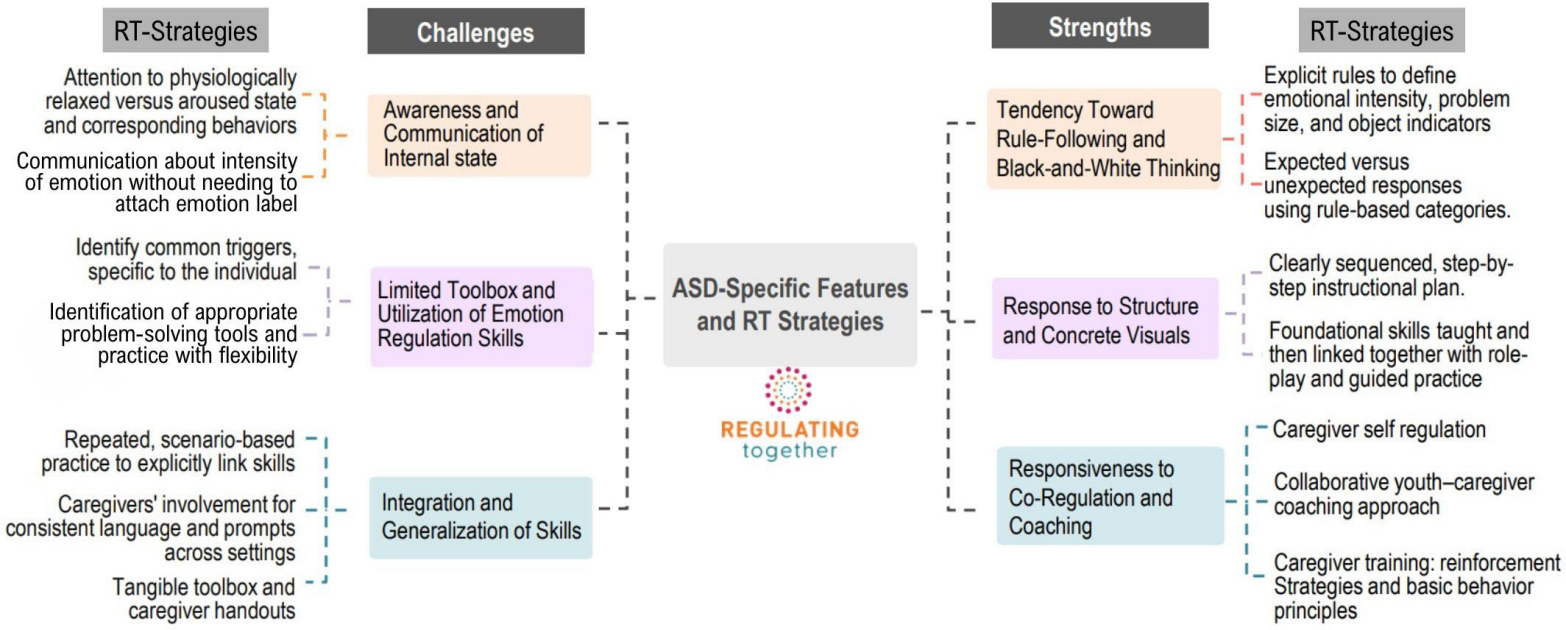
ASD-specific features and RT strategies used to inform intervention design.

**Figure 2:**
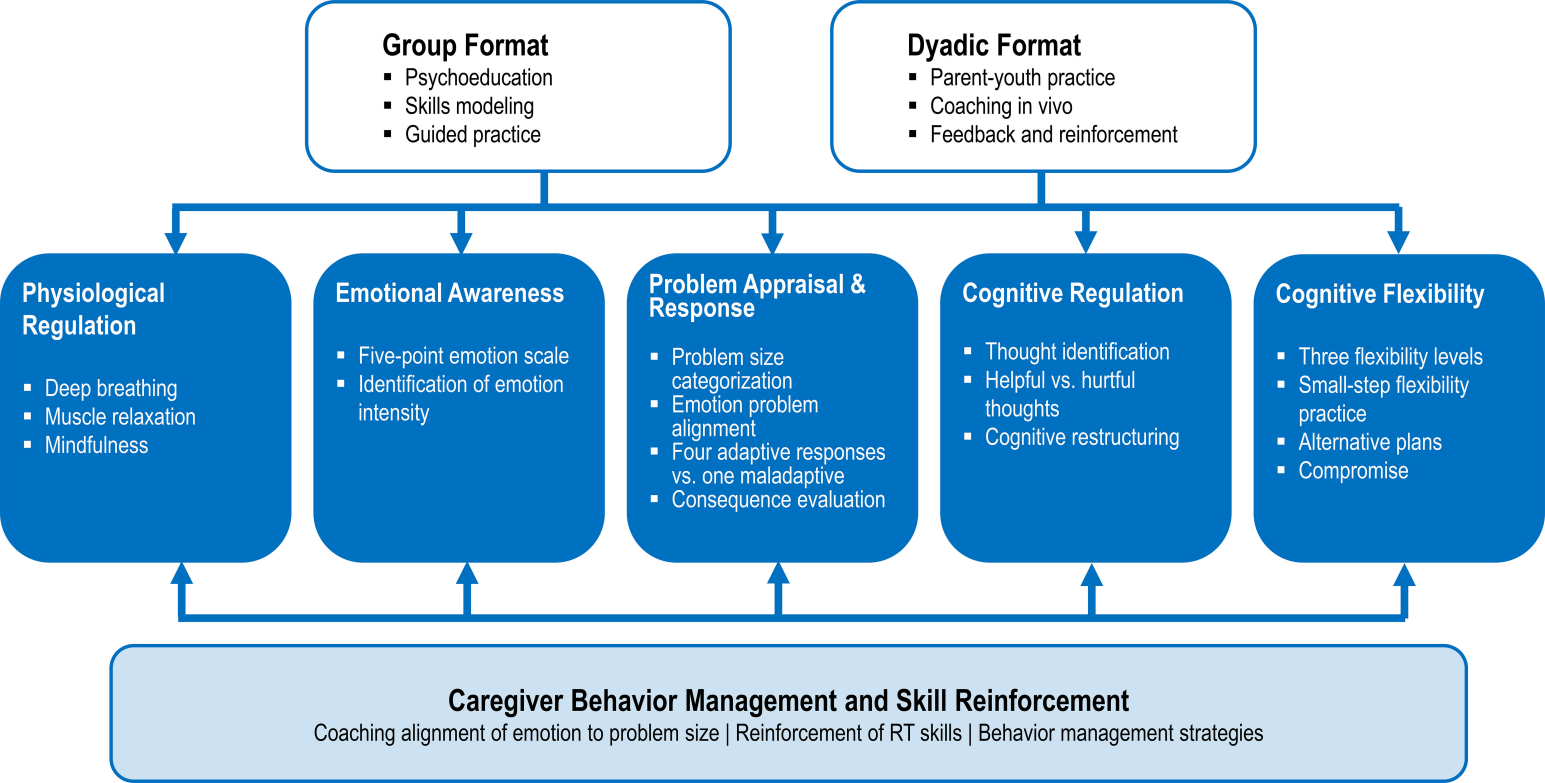
RT delivery and skill domains.

This highlights the need for interventions designed to enhance ER skills, which led to the development of Regulating Together (RT; (10)), a manualized group intervention targeting ER difficulties in autistic youth that incorporates active caregiver involvement. RT is a group based intervention that meets either twice weekly or once weekly with both caregiver and child groups that meet separately. Caregivers are taught behavior management skills and how to coach their children in using the skills taught in the program. RT aims to increase overall ER through the following strategies and skills: improving emotional awareness/insight through rating emotions on a 5-point scale, regulation of physiological states through breathing, muscle relaxation and mindfulness, understanding of problem sizes with a concrete, easily applicable model, problem-solving incorporating solutions and acceptance-based responses, cognitive identification of helpful and unhelpful thoughts and restructuring of unhelpful thoughts, and flexibility through practice and use of a bonus plan as a means of increasing overall ER. Teaching approaches for these skills include *in vivo* practice, sorting activities, visual supports, application through scenarios and real-life examples, and assigned home practice. Caregivers also learn how to manage their own emotions and utilize the skills in the program for themselves and their children. In doing so, RT also supports skill-building in areas of importance for autistic individuals that may not be as directly targeted in other existing interventions. RT has been refined through clinical implementation with autistic children and their caregivers, informed by caregiver feedback and clinical observation. In the discussion that follows, RT treatment strategies are utilized as an exemplar for how to address common challenges for autistic youth with ER skill difficulties. Additionally, strengths common to autism are shared with strategies for the purpose of building on these strengths to foster ER skill development in autistic youth. See **Figure 1** for a breakdown of RT strategies addressing the highlighted strengths and challenges. Please see **Figure 2** for an outline of the RT intervention format and skills.

## 2 Potential challenges

### 2.1 Challenge 1: internal awareness and communication of internal state

An essential ER skill is the ability to change/reduce emotionally related physiological arousal. Multiple relaxation strategies are efficacious for modulating physiological responses, including deep breathing, progressive muscle relaxation, and mindfulness-based interventions. To address challenges specific to autistic populations, RT utilizes visuals and active components of relaxing such as tracing while breathing. Concrete, active breathing and muscle relaxation strategies in child friendly language may be better received, including Figure 8 Breathing or Squeezing Oranges. By creating fun, engaging yet simple relaxation experiences, practice is more likely to continue. To further develop recognition of internal physiological states, RT uses sorting activities to help differentiate between the physiological states of relaxation and arousal. Particular attention is paid to heartrate, breathing, muscle tension/posture, facial expressions, stomach sensations, thought patterns, and actions. Symptoms of aroused states are highlighted as ways to recognize when triggered.

Anecdotally, many parents describe their autistic children as “going from 0 to 60,” suggesting rapid escalation of emotions. RT therefore focuses on increasing affective awareness around initial escalation of emotions before the emotional response becomes too intense to manage or change its trajectory. RT scales emotion levels with objective indicators based on physiological experiences and typical behaviors. For example, Level 4 indicates feeling anxious, angry, or sad with elevated arousal (e.g., faster breathing, upset stomach) and behaviors such as stomping or withdrawing. Objectively anchored scales help individuals contextualize emotions and triggers, allowing for prescriptive guidance, including when to use skills (e.g., “use your skills as soon as you reach Level 3” (feeling triggered)).

Emotional experiences for autistic individuals may also be more ambiguous, with multiple emotions often occurring concurrently (e.g., anxious and angry). Many individuals therefore experience difficulty identifying or labeling specific emotions. To address this, RT utilizes broad, objective labels that do not require specification of which emotion(s) is being experienced but instead indicate emotional *intensity*. When combined with objective indicators of problem sizes (as small, medium, and large), children can more easily match appropriate levels of emotion to the size of the problem, creating a clear goal for the emotional trajectory. For instance, during smaller problems, a goal would be to maintain or reduce to a low level of emotion (i.e., Levels 1-3). To support perspective taking while still validating the child’s emotional experiences and building on child strengths in concrete thinking (discussed in more detail below), RT coaches sizing of problems, separate from emotional experiences, by using objective indicators that may also aid perspective taking. Skill utilization in RT is based on *how much* emotion (intensity) and *how objectively big* the problem is (problem size) without having to parse the prevailing emotions.

### 2.2 Challenge 2: limited toolbox and utilization of emotion regulation skills

Many caregivers report their children identify and even effectively communicate *about* ER skills yet have difficulty utilizing skills independently when needed. Even when skill use is still accessible at lower levels of emotion (e.g., 1 or 2 levels), most individuals struggle to *effectively* apply skills that are new or rarely utilized. Initiating and utilizing skills is less effortful when muscle memory has been built through extensive regular practice and caregiver modeling of calming strategies. Therefore, RT emphasizes regular practice of coping skills by beginning each session with structured relaxation or mindfulness practice. Outside of sessions, families are encouraged to practice relaxation and mindfulness frequently (e.g., daily and during moments of calm), to build mastery. Once skills are established, skills can be more effectively utilized in moments of building or high distress. Over time, regular relaxation practice may also help lower levels of physiological arousal (e.g., (11)).

Caregivers often take charge of problem-solving or attempt to prevent problems for autistic youth. As such, they may have fewer opportunities for independent problem-solving practice and have limited problem-solving strategies. Problem-solving is critical for ER by enabling individuals to analyze a situation, identify the problem, and choose an effective action to reduce stress. However, many problems do not have clear solutions or knowable outcomes, which can be especially challenging for autistic individuals due to intolerance of uncertainty. RT provides prescriptive options for problem-solving using structured visuals with concrete examples. RT starts with simple, scenario-based problems in which children practice a problem-solving model to assess situations, reappraise them, and alter feelings, ultimately leading to changes in behavior. RT stresses that, at times, some problems are not easily solved and problem-solving comes down to letting the problem go and moving on or letting the problem bother you.

Underscoring the benefits of moving on may not be enough motivation to get unstuck and let it go, especially for autistic youth. RT outlines strategies for getting unstuck and moving on alongside benefits of relaxation and increasing cognitive flexibility. In addition to problem-solving and moving-on options, mindfulness-based skills are taught to refocus attention, increase nonjudgmental awareness, and decrease stress. Mindfulness-based interventions have, themselves, been demonstrated to promote ER in autism (12, 17). In addition, RT prescribes methods for practicing and increasing cognitive flexibility. Participants are taught to identify levels of flexibility, develop contingency plans, practice compromise, and find small flexing opportunities, such as trying something new. RT encourages participants to ask helpful questions before the behavior, including “can anything good happen because of this change?”.

### 2.3 Challenge 3: integration and generalization of skills

Even after proficiency has been achieved for individual skills, ER is a complicated, multi-faceted process best achieved by combining these skills. Specifically, differences in executive functioning and cognitive flexibility may increase the need for repetition and multimodal instruction (13). RT provides instruction and increased, repetitive practice through scenario-based exercises so that autistic children learn how each skill connects to and informs other skills (e.g., the size of a problem informs the appropriateness of emotional level and problem-solving options, or modulating emotional intensity may increase flexibility). Additionally, caregivers are provided with materials to directly coach children on skill integration. Caregiver involvement is key by creating a common language across therapy and home for identifying/prompting skills and communicating about emotion. To promote consistency across multiple systems and settings, RT encourages caregivers to share this common language and strategies with educators and other services providers to ensure coordinated support in implementing the strategies.

At the end of RT intervention, participants generate a tangible, physical toolbox which also serves as a way to reinforce skill generalization. The toolbox contains visual representations of skills, which reinforces interconnectivity of skills. This represents the metaphorical “toolbox” participants created by learning and connecting skills throughout the group.

## 3 Autism specific strengths

### 3.1 Strength 1: tendency toward rule-following and black-and-white thinking

The literature suggests autistic individuals have a propensity to demonstrate rule-governed behavior more consistently; once a rule is established, maintenance of rules may also be more sustained in this population (14). In addition, autistic youth tend to demonstrate more dichotomous (black-and-white) thinking (14, 15). Jointly, these characteristics function as important strengths for youth when they are building a new regulatory framework, by allowing them to focus on details and systems and thriving in a highly structured learning environment.

In fact, caregivers often report in therapy that their autistic children respond well to concrete rules or frameworks in therapy such as the 5-point scale or size of the problem curriculum. Complex ideas, such as emotional intensity and emotional responses that match the situation, can be made more concrete with clear “rules,” by reducing uncertainty, which allows for more rapid skill acquisition, enhanced skill retention, and engagement with the rule-based concepts. RT capitalizes on this strength by providing clearly defined guidelines for determining intensity of emotions, problem sizes, and response to problems. For instance, RT teaches families to use a 5-point scale of emotions with clear behavioral and physiological indicators to understand and communicate about an emotional experience. Rules for when to use coping skills can be conveyed based on this scale (e.g., RT recommends using coping skills as soon as one reaches Level 3 or when a trigger occurs and that caregivers respond differently when children are at lower levels versus high levels).

Dichotomous thinking as well as rule-following tendencies may help autistic youth with the ability to differentiate objectively between categories of problem sizes (small, medium, or big in RT) rather than rely on feelings about their size and may aid in acquisition and maintenance of appropriate emotional responses for various situations. Categorization of appropriate/expected behaviors versus inappropriate/unexpected behaviors has regularly been utilized to address target behaviors for autistic youth, due in part to this strength. RT utilizes this approach through its categorization of problem-solving responses (described above). These tools are provided in both verbal and visual formats with clear and concrete examples, such that they can be applied in a more literal way that is commonly observed in autism.

### 3.2 Strength 2: response to structure and concrete visuals

The skills within RT build upon themselves and create a step-by-step structured plan for autistic youth and their caregivers to follow to build ER and decrease emotion dysregulation prior to triggering events and during difficult emotion states. While this type of plan is helpful for most children, it is especially helpful for autistic youth given their strength in following structure and clearly defined plans. Autistic youth may utilize these scripted structures and plans more readily as they provide predictability and certainty, which autistic youth often seek out (16, 18).

This emphasis on structure is reflected in the overall RT program and session format, including visual schedules that outline session sequences, which support sustained engagement. Families are coached on providing structured expectations for skill practice. New skills are linked to previously taught skills as well as direction of use. For instance, children are instructed on coping skills prior to triggers, but coping skills are subsequently highlighted as an appropriate response to a trigger. Similarly, the 5-point scale of emotional intensity, problem sizes, and problem solving are all taught as separate skills for independent practice.

Once foundation skills are developed/established, families are then provided with a structure for integrating these skills: the size of a problem should dictate the appropriate level of emotion, which children may be aided in achieving though use of coping skills, then children should respond with one of the four appropriate problem-solving skills. The deliberate review and anchoring of new skills to previously learned skills may further increase the propensity of youth to respond with this healthier regulatory process in the future. In a step-by-step framework, including role-play, repeated practice, and coaching questions that prompt skill use in a predictable sequence RT leverages the positive response to structure in this population to help consolidate the integrated skills. Structuring the intervention in this way allows the youth and their families to know what to expect and how to follow plans which further strengthen engagement and increase buy-in.

### 3.3 Strength 3: responsiveness to co-regulation and coaching

Autistic youth often demonstrate a strength of co-regulation with caregivers. Although many would view this as a weakness and a sign that youth cannot regulate independently, we view this as a valuable strength as it allows caregivers to become coaches in regulation development and increases in-the-moment implementation of skills. The collaborative approach of youth and coach utilized in RT builds upon this strength by giving caregivers the skills they need to more effectively regulate both themselves and their youth as well as help the child begin to build more independent regulation. The promotion of collaboration in RT also encourages the youth to apply skills more broadly throughout the day for both themselves and others such as caregivers and other family members. By externalizing and involving the whole family in the process, additional practice and application is achieved.

Based on this collaborative framework, The RT model necessitates caregivers to learn alongside their children, such that regulation becomes a team effort. When caregivers deepen their understanding of ER skills, they can more easily model, prompt, assist, and reinforce skill use in real-life situations. Modeling by caregivers may motivate and normalize skill use for children. Children can practice skills with, or on, caregivers when it may be difficult to start for themselves (e.g., helping a caregiver size their own problem), which may reduce initiation demands. Indeed, caregiver informed clinical perspectives developed during active RT treatment suggest autistic youth may make progress through first applying skills to another person before applying those same skills to themselves. RT devotes time in caregiver sessions to provide psychoeducation and help families develop reinforcement systems as well as general behavior principles that frame and reinforce skill use. Importantly, active caregiver engagement allows for continuous learning and skill practice supported by caregivers even after formal intervention has concluded.

## 4 Discussion

Given its transdiagnostic nature, intervening on emotion dysregulation may benefit children and adolescents with a broad range of psychopathology but may require adaptation for autistic individuals. Proposed solutions for barriers to improving ER in autism can provide guidance for clinicians working with autistic youth regardless of experience with this population. Additionally, autistic youth have a number of unique traits that can be capitalized upon as strengths to support development of ER. While many of these characteristics have been previously identified in the literature and through clinical experience, comprehensive options for increasing ER skills, with consideration of autism-specific traits, remain limited. Interventions, such as RT, which target multiple facets of ER, provide broader insight into the utility of specific methods for addressing ER in autism. We suggest that both the skills themselves and the modality through which intervention is delivered are essential to drive change in a way that utilizes strengths and addresses unique challenges. It is necessary to continue researching the effectiveness of skill development for reducing emotion dysregulation in autism, examining the impact of autism-specific factors on building ER acquisition. Additionally, future studies should determine whether these proposed solutions sufficiently address specific challenges for this population and evaluate how well interventions for this population address the diverse needs of autistic individuals and families.

## Data Availability

The original contributions presented in the study are included in the article/supplementary material. Further inquiries can be directed to the corresponding author.
